# Risk Factors Associated With Long-Term Hospitalization in Patients With COVID-19: A Single-Centered, Retrospective Study

**DOI:** 10.3389/fmed.2020.00315

**Published:** 2020-06-09

**Authors:** Yiqun Wu, Bingbo Hou, Jielan Liu, Yingying Chen, Ping Zhong

**Affiliations:** ^1^Department of Respiratory Section II, The Third Hospital of Xiamen Affiliated to Fujian University of Traditional Chinese Medicine, Xiamen, China; ^2^Department of Cardiology, Zhongshan Hospital Affiliated to Xiamen University, Xiamen, China; ^3^Department of Internal Medicine, Xiamen Lotus Hospital, Xiamen, China; ^4^Department of Cardiac and Cerebral Fiction, Xiamen Xian Yue Hospital, Xiamen, China; ^5^BE and Phase i Clinical Trial Center, The First Affiliated Hospital of Xiamen University, Xiamen, China

**Keywords:** coronavirus disease 2019, SARS-Cov-2, prognosis, recovery time, electrolyte imbalance

## Abstract

**Background:** The coronavirus disease 19 (COVID-19) pandemic has become a global threat. Few studies have explored the risk factors for the recovery time of patients with COVID-19. This study aimed to explore risk factors associated with long-term hospitalization in patients with COVID-19.

**Methods:** In this retrospective study, patients with laboratory-confirmed COVID-19 hospitalized in a hospital in Wuhan by March 30, 2020, were included. Demographic, clinical, laboratory, and radiological data from COVID-19 patients on hospital admission were extracted and were compared between the two groups, defined as short- and long-term hospitalization, respectively according to the median hospitalization time. Univariable and multivariable logistic regression methods were performed to identify risk factors associated with long-term hospitalization in patients with COVID-19.

**Results:** A total of 125 discharged patients with COVID-19 were reviewed, including 123 general patients and two severe patients. The median hospitalization time was 13.0 days (IQR 10.0–17.0). Among them, 66 patients were discharged <14 days (short-term group) and 59 patients were discharged ≥14 days (long-term group). Compared with the short-term group, patients in the long-term group had significantly higher levels of C-reactive protein (*P* = 0.000), troponin I (*P* = 0.002), myoglobin (*P* = 0.037), aspartate aminotransferase (*P* = 0.005), lactic dehydrogenase (*P* = 0.000), prothrombin time (*P* = 0.030), fibrinogen (*P* = 0.000), and D-dimer (*P* = 0.006), but had significantly lower levels of lymphocyte count (*P* = 0.001), platelet count (*P* = 0.017), albumin (*P* = 0.001), and calcium (*P* = 0.000). Additionally, the incidences of hypocalcemia (*P* = 0.001), hyponatremia (*P* = 0.021), hypochloremia (*P* = 0.019), and bilateral pneumonia (*P* = 0.000) in the long-term group were significantly higher than those in the short-term group. Multivariable regression showed that hypocalcemia (*P* = 0.007, OR 3.313, 95% CI 1.392–7.886), hypochloremia (*P* = 0.029, OR 2.663, 95% CI 1.104–6.621), and bilateral pneumonia (*P* = 0.009, OR 5.907, 95% CI 1.073–32.521) were independent risk factors associated with long-term hospitalization in patients with COVID-19. Furthermore, a ROC curve where the area under the ROC was 0.766 for retained variables is presented.

**Conclusions:** Hypocalcemia, hypochloremia, and bilateral pneumonia on hospital admission were independent risk factors associated with long-term hospitalization in patients with COVID-19. To the best of our knowledge, this is the first study to highlight the importance of electrolyte imbalance in predicting the hospitalization time of patients with COVID-19.

## Introduction

Coronavirus disease 2019 (COVID-19), caused by severe acute respiratory syndrome coronavirus-2 (SARS-Cov-2), continues spreading rapidly worldwide. Globally, more than five million cases including 350, 000 deaths were confirmed with COVID-19 by May 28, 2020 (1). The clinical spectrum of patients with COVID-19 is quite broad, ranging from mild symptoms such as simple cold to severe illness. Most reported cases only experienced mild or moderate symptoms (2–4). However, one previous study indicated that 15.7% of patients with COVID-19 developed a severe illness after admission to a hospital (2). Moreover, some patients could develop severe pneumonia, acute respiratory distress syndrome (ARDS), and even multiple organ failure during hospitalization (5, 6). Thus, early management is thought to be an essential strategy for the prevention and management of COVID-19 (7).

Wuhan, the epicenter of the COVID-19 outbreak in China, was struggling to cope with the COVID-19. Especially, healthcare systems were facing extreme pressure. Tens of thousands of healthcare workers from across the country were then rapidly mobilized to different hospitals in Wuhan (8, 9). To date, not so many studies have been reported regarding clinical characteristics and outcomes of patients with COVID-19 admitted and treated in these hospitals. Moreover, much more interests were mainly focused on the clinical course and the outcomes of severe or critical patients with COVID-19 (3–6, 10), while information on the outcomes of non-severe patients is still limited. Furthermore, as no medication with definite therapeutic effects were available, symptomatic treatment was the main therapeutic strategy in COVID-19 patients during hospitalization. Given this, the recovery time of patients with COVID-19 is probably dependent on the patient's immunity (11). To date, several studies have reported the hospitalization time of COVID-19 patients with different severities (4, 10, 12–16). However, to the best of our knowledge, no study has been reported aiming to explore risk factors for the hospitalization time of patients with COVID-19.

To combat with the COVID-19 outbreak in Wuhan, a team of 138 medical workers and professionals from Xiamen city (1, 000 kilometers away from Wuhan) was mobilized to help and work on E3-9 ward in Wuhan Tongji Hospital Guanggu Branch from February 10, 2020, to March 30, 2020. As members of them, here, we present information of patients with laboratory-confirmed COVID-19 admitted to the E3-9 ward during this period. The purpose of this study was to investigate the clinical characteristics and laboratory and radiological results of hospitalized COVID-19 patients, with a special focus on exploring risk factors associated with long-term hospitalization in patients with COVID-19.

## Methods

### Study Population and Data Collection

COVID-19 patients admitted to the E3-9 ward in Wuhan Tongji Hospital Guanggu Branch between February 10, 2020, and March 30, 2020, were included. Patients included in this study were clinically diagnosed as “COVID-19,” HYPERLINK “http://www.nhc.gov.cn/” and the diagnosis of COVID-19 in all patients was confirmed by detecting SARS-CoV-2 RNA in pharyngeal swab samples using a virus nucleic acid detection kit in the clinical laboratory of Tongji Hospital based on the “diagnosis and treatment scheme for COVID-19 of China” from the National Health Commission of the People's Republic of China (http://www.nhc.gov.cn/). This study was approval by the Ethics Committee of The Third Hospital of Xiamen Affiliated to Fujian University of Traditional Chinese Medicine and informed consent was waived by the Ethics committee for this retrospective study.

Demographic information and clinical medical records (clinical characteristics, laboratory and radiological results) from COVID-19 patients on hospital admission were extracted and retrospectively analyzed. Clinical characteristics included symptoms onset (e.g., fever, cough), the time from illness onset to hospital admission, vital signs on hospital admission (heart rate, blood pressure, temperature, pulse oximeter O_2_ saturation), comorbidities (e.g., hypertension, type 2 diabetes, coronary heart disease). Laboratory results (white blood cell, lymphocyte count, hemoglobin, platelet count, C-reactive protein, high sensitive troponin I, B-type natriuretic peptide, myoglobin, creatine kinase isoenzyme, albumin, alanine aminotransferase, aspartate aminotransferase, urea, creatinine, lactic dehydrogenase, potassium, sodium, calcium, chlorine, prothrombin time, activated partial thromboplastin time, thrombin time, fibrinogen, and D-dimer) and radiological findings (chest computed tomography (CT) scan) were collected.

The severity of COVID-19 on hospital admission and treatments during patients' hospitalization (oxygen therapy, antibacterial agents, antiviral agents) were also collected. According to the “diagnosis and treatment scheme for COVID-19 of China,” the severity of COVID-19 was categorized as general, severe, or critical. The general type represents patients with non-pneumonia and mild to moderate pneumonia. The severe type was characterized by (1) dyspnea (respiratory frequency ≥30 rates per minute); (2) blood oxygen saturation ≤93%; (3) PaO2/FiO2 ratio <300, and/or lung infiltrates >50% within 24–48 h (satisfying at least one of the above items). However, since the short outcome was discharge, deaths and the patients transferred to other designated hospitals were not included in this study.

### Risk Factors for Long-Term Hospitalization

According to the “diagnosis and treatment scheme for COVID-19 of China,” criteria of being discharged from hospital for COVID-19 patients in this ward were (1) The body temperature returned to normal for more than 3 days; (2) The respiratory symptoms recovered significantly; (3) The acute exudative lesions showed in chest CT improved significantly; (4) A negative result of SARS-Cov-2 detected by RT-PCR was observed in two consecutive respiratory tract samples (at least 24 h apart). Besides, the recovery situation at least 2 weeks after hospital discharge was followed and recorded in discharged patients through a regional management system for COVID-19. According to the median hospitalization time, patients included in the present study were divided into two groups: short-term group and long-term group, respectively. Demographic, clinical, laboratory, and treatment data in the two groups were compared and risk factors for long-term hospitalization were identified.

### Statistical Analysis

The data were analyzed by SPSS statistic 22.0 (SPSS Inc., Chicago, USA). Continuous variables were expressed as median and interquartile range (IQR), and the differences between the two groups were analyzed using the Mann-Whitney U test. Categorical values were expressed as frequencies, and the differences between the two groups were analyzed using χ^2^ test or Fisher's exact test. To further explore the risk factors associated with long-term hospitalization, univariable and multivariable logistic regression models were performed. Candidate variables with a *P* ≤ 0.10 in univariable analysis were included in the multivariable model and a stepwise forward selection was performed. However, the variables highly related to the outcome (e.g., age, comorbidity) were also considered in this model, even *P* > 0.1 for these variables (10, 17). In order to examine whether these retained variables could be predictive for long-term hospitalization in this model, the Hosmer and Lemeshow test and a receiver operating characteristic curve (ROC curve) were performed as well. All statistical significant difference was defined as *P* < 0.05.

## Results

### Comparisons of Demographic and Clinical Characteristics of 125 Patients With COVID-19 Between the Two Groups

A total of 139 patients with laboratory-confirmed COVID-19 were admitted to our ward from February 10, 2020, to March 30, 2020. Among them, three patients died during hospitalization, and 11 patients were transferred to other designated hospitals. Finally, 125 patients were discharged from our ward by Mar 30, 2020, and were followed to confirm that they were still in recovery state 2 weeks after discharge. As of April 15, 2020, no discharged patient was recorded to have a positive result of SARS-Cov-2 in pharyngeal swab samples in the 125 patients. Accordingly, clinical records of 125 discharged patients with COVID-19 were reviewed in this study. The median age of 125 patients was 55.0 years (IQR 40.0–68.5), and 53 patients (53/125; 42.40%) were over 60 years old. Among them, 63 patients (63/125; 50.40%) had one or more comorbidities. Hypertension, type 2 diabetes, and coronary heart disease were the most common comorbidity. The median time from illness onset to hospital admission was 15.0 days (IQR 7.0–30.0), while the median time from illness onset to hospital discharge was 30.0 days (IQR 21.5–43.0). The most frequent symptoms of illness onset were fever (70/125; 56.00%) and cough (64/125; 51.20%), but only 12.80% (16/125) of patients had a fever on admission. In this study, 98.40% (123/125) of the patients were categorized as general type, while only two patients (2/125; 1.60%) were categorized as severe type.

**Figure 1:**
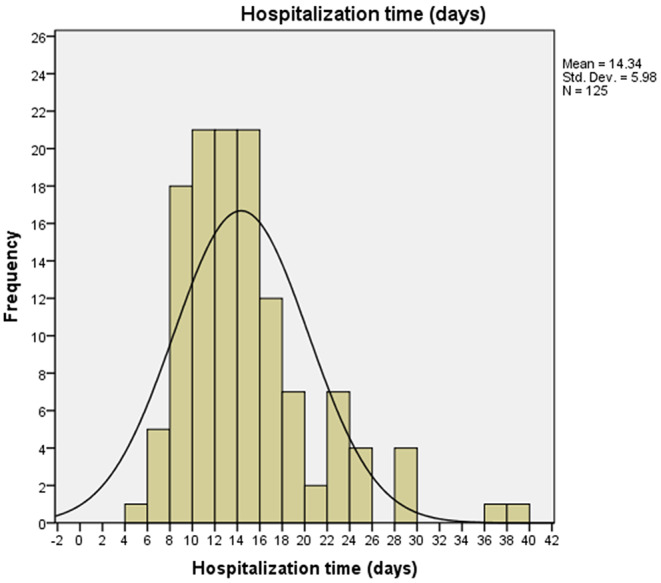
Distribution of hospitalization time of 125 patients with COVID-19. The median hospitalization time was 13.0 days (IQR 10.0–17.0), ranging from 5.0 to 39.0 days.

The median hospitalization time was 13.0 days (IQR 10.0-17.0), ranging from 5.0 days to 39.0 days (Figure 1). Thus, 66 patients were discharged <14 days (short-term group) and 59 patients were discharged ≥14 days (long-term group). Comparisons of demographic and clinical characteristics of 125 patients with COVID-19 between the two groups are shown in Table 1. The median diastolic blood pressure in the short-term group was significantly higher than that in the long-term group (*P* = 0.022), while no differences were observed in heart rate, systolic blood pressure, temperature, and pulse oximeter O_2_ saturation between the two groups. In addition, there were no significant differences in age, sex, the time from illness onset to hospital admission, comorbidity, and the onset symptoms between the two groups.

**Table 1:** Comparisons of demographic and clinical characteristics of 125 patients with COVID-19 between the two groups.

**Characteristics**	**Total (*n =* 125)**	**Short-term group (*n =* 66)**	**Long-term group (*n =* 59)**	***Z*/χ^2^**	***P***
Age (years)	55.0 (40.0–68.5)	52.50 (37.0–66.0)	59.0 (41.0–70.0)	−1.652	0.098
<60	72 (57.60)	42 (63.64)	30 (50.84)	2.086	0.149
≥60	53 (42.40)	24 (36.36)	29 (49.16)		
**Sex**					
Male	66 (52.80)	33 (50.00)	33 (55.93)	0.440	0.507
Female	59 (47.20)	33 (50.00)	26 (44.07)		
**Date of illness onset**					
Jan or earlier	51 (40.80)	24 (36.36)	27 (45.76)	1.139	0.286
Feb or later	74 (59.20)	42 (63.64)	32 (54.24)		
Time form illness onset to admission (days)	15.0 (7.0–30.0)	16.0 (7.5–31.75)	12.0 (7.0–21.0)	−1.519	0.129
≤ 15	65 (52.00)	29 (43.94)	36 (61.02)	3.640	0.056
>15	60 (48.00)	37 (56.06)	23 (38.98)		
**Comorbidity**					
Hypertension	35 (28.00)	22 (33.33)	13 (22.03)	1.973	0.160
Type 2 diabetes	25 (20.00)	13 (19.70)	12 (20.34)	0.008	0.929
Coronary heart disease	11 (8.80)	4 (6.06)	7 (11.96)	1.307	0.253
≥1 comorbidity	63 (50.4)	34 (51.52)	29 (49.15)	0.070	0.792
**Medications**					
0	78 (62.40)	43 (65.15)	35 (59.32)		
1–2	26 (20.80)	14 (21.21)	12 (24.34)	1.014	0.602
≥3	21 (16.80)	9 (13.64)	12 (24.34)		
**Onset symptoms**					
Fever	70 (56.00)	36 (54.54)	34 (57.63)	0.120	0.729
Cough	64 (51.20)	37 (56.06)	27 (45.76)	1.322	0.250
Temperature (°C)	36.5 (36.3–36.8)	36.5 (36.3–36.8)	36.6 (36.4–36.6)	−0.706	0.480
>37.3	16 (12.80)	8 (12.12)	8 (13.56)	0.056	0.810
Heart rate (beats/min)	90 (78.5–101.0)	90.0 (80.0–102.25)	90 (78.0–100.0)	−0.381	0.703
>100	31 (24.80)	17 (25.76)	14 (23.73)	0.069	0.793
SBP (mm Hg)	130.0 (130.0–141.0)	130.5 (119.5–149.5)	127.0 (119.0–136.0)	−1.653	0.098
≥140	37 (29.60)	27 (40.91)	10 (16.95)	8.582	0.003^*^
DBP (mm Hg)	80.0 (73.0–90.0)	82.0 (74.0–95.0)	80.0 (70.0–87.0)	−2.289	0.022^*^
≥90	33 (26.40)	24 (36.36)	9 (15.25)	7.144	0.008^*^
SPO_2_ (%)	98.0 (96.5–98.5)	98.0 (97.0–99.0)	97.0 (96.0–98.0)	−1.401	0.161
**Clinical type on admission**					
Severe	2 (1.60)	1 (1.52)	1 (1.69)	0.006	1.000
General	123 (98.40)	65 (98.48)	58 (98.31)		

Data are shown as median (interquartile range) or n (%). P-values were calculated by Mann-Whitney U test, χ^2^ test or Fisher's exact test, as appropriate. SBP, systolic blood pressure; DBP, diastolic blood pressure; SPO_2_, pulse oximeter O_2_ saturation;

* *P < 0.05 denoted significant difference between patients with short-term and long-term hospitalization*.

### Comparisons of Laboratory Indices, Radiographic Findings, and Treatments of 125 Patients With COVID-19 Between the Two Groups

Lymphocytopenia occurred in 43 patients (43/125; 34.40%), and anemia was observed in 61 patients (61/125; 48.80%). An elevated level of C-reactive protein was found in 98 patients (98/125; 78.40%), in which 57 patients (57/125; 45.60%) had a level over 10 mg/L. Meanwhile, elevated levels of alanine aminotransferase (22/125; 17.60%), aspartate aminotransferase (18/125; 14.40%), urea (6/125; 4.80%), creatinine (11/125; 8.80%), lactic dehydrogenase (51/125; 40.80%), fibrinogen (35/125; 28.00%), and D-dimer (50/125; 40.00%) were also observed in the 125 patients. A large proportion of patients had electrolyte imbalance including hypocalcemia (81/125; 64.80%), hypokalemia (13/125; 10.40%), hyponatremia (22/125; 17.60%), and hypochloremia (38/125; 30.40%). In addition, there were 61 patients (61/125; 48.80%) with bilateral pneumonia, 53 patients (53/125; 42.40%) with unilateral pneumonia, and 11 patients (11/125; 8.80%) without pneumonia.

Comparisons of laboratory indices, radiographic findings, and treatments of 125 patients with COVID-19 between the two groups are presented in Table 2. Compared with patients in the short-term group, patients in the long-term group had significantly higher levels of C-reactive protein (*P* = 0.000), high sensitive troponin I (*P* = 0.002), myoglobin (*P* = 0.037), aspartate aminotransferase (*P* = 0.005), lactic dehydrogenase (*P* = 0.000), prothrombin time (*P* = 0.030), fibrinogen (*P* = 0.000), and D-dimer (*P* = 0.006), but had significantly lower levels of lymphocyte count (*P* = 0.001), platelet count (*P* = 0.017), albumin (*P* = 0.001), and calcium (*P* = 0.000). In addition, the incidences of hypocalcemia (*P* = 0.001), hyponatremia (*P* = 0.021), and hypochloremia (*P* = 0.019) in the long-term group were significantly higher than those in the short-term group. Patients in the long-term group had a higher incidence of bilateral pneumonia and a lower incidence of unilateral pneumonia compared with patients in the short-term group (*P* = 0.000). Patients in the long-term group were more likely to receive antibiotics treatment (*P* = 0.001).

**Table 2:** Comparisons of laboratory indices, radiographic findings, and treatments of 125 patients with COVID-19 between the two groups.

**Characteristics**	**Total (*n =* 125)**	**Short-term group (*n =* 66)**	**Long-term group (*n =* 59)**	***Z*/χ^2^**	***P***
WBC (×10^9^/L)	5.90 (4.54–7.43)	5.87 (4.49–7.42)	5.90 (4.56–7.58)	−0.040	0.968
<3.5	13 (10.40)	6 (9.09)	7 (11.86)	2.467	0.291
3.5–9.5	100 (80.00)	56 (84.84)	44 (74.58)		
>9.5	12 (9.60)	4 (6.07)	8 (13.56)		
Lymphocyte count (×10^9^/L)	1.31 (0.87–1.82)	1.59 (1.07–2.09)	1.18 (0.76–1.58)	−3.326	0.001^*^
<1.1	43 (34.40)	18 (27.27)	25 (42.37)	4.540	0.103
1.1–3.2	81 (64.80)	48 (72.73)	33 (55.93)		
>3.2	1 (0.80)	0 (0)	1 (0.80)		
Hemoglobin (g/L)	130.0 (118.0–140.0)	133.0 (120.0–144.0)	128.0 (116.0–137.0)	−1.234	0.217
Anemia	61 (48.80)	29 (43.94)	32 (54.24)	1.322	0.250
PLT (×10^9^/L)	210.0 (170.0–257.0)	223.5 (187.75–270.75)	196.0 (160.0–243.0)	−2.396	0.017^*^
<125	6 (4.80)	1 (1.52)	5 (8.47)	3.321	0.190
125–350	103 (82.40)	56 (84.85)	47 (79.66)		
>350	16 (12.80)	9 (13.63)	7 (11.87)		
CRP (mg/L)	6.70 (1.30–41.8)	2.20 (0.68–12.82)	17.40 (2.80–66.50)	−4.044	0.000^*^
<1	27 (21.60)	20 (30.30)	7 (11.86)	18.931	0.001^*^
1–3	24 (19.20)	16 (24.24)	8 (13.56)		
3–10	17 (13.60)	11 (16.67)	6 (10.17)		
10.1–50	30 (24.00)	13 (19.70)	17 (28.81)		
>50	27 (21.60)	6 (9.10)	21 (35.59)		
Troponin I (pg/mL)	3.9 (1.9–10.3)	1.90 (1.90–7.52)	6.90 (1.90–12.50)	−3.043	0.002^*^
≤ 34.2	115 (92.00)	63 (95.45)	52 (88.14)	2.267	0.189
>34.2	10 (8.00)	3 (4.55)	7 (11.86)		
BNP (pg/mL)	65.0 (23.0–178.0)	52.0 (19.75–178.0)	96.0 (29.0–209.0)	−1.328	0.184
<486	107 (85.60)	58 (87.88)	49 (83.05)	0.589	0.443
≥486	18 (14.40)	8 (12.12)	10 (16.95)		
Myoglobin (ng/mL)	35.0 (27.7–75.65)	32.70 (24.52–53.18)	40.30 (29.0–102.5)	−2.087	0.037^*^
≤ 154.9	111 (88.80)	61 (92.42)	50 (84.75)	1.874	0.174
>154.9	14 (11.20)	5 (7.58)	9 (15.25)		
CKMB (ng/mL)	0.7 (0.5–1.2)	0.70 (0.5–1.2)	0.8 (0.5–1.2)	−0.583	0.560
Albumin (g/L)	40.0 (33.75–43.15)	41.3 (36.1–44.43)	37.4 (31.9–41.9)	−3.375	0.001^*^
35–52	85 (68.00)	42 (78.79)	33 (55.93)	7.479	0.006^*^
<35	40 (32.00)	14 (21.21)	26 (44.07)		
ALT (U/L)	24.0 (14.0–36.5)	20.50 (12.50–34.50)	25.0 (18.0–38.0)	−1.363	0.173
≤ 41	103 (82.40)	55 (83.33)	48 (81.36)	0.084	0.772
>41	22 (17.60)	11 (16.67)	11 (18.64)		
AST (U/L)	22.0 (16.0–32.5)	19.00 (14.0–26.25)	26.0 (18.0–36.0)	−2.797	0.005^*^
≤ 40	107 (85.60)	58 (87.88)	49 (83.05)	0.589	0.443
>40	18 (14.40)	8 (12.12)	10 (16.95)		
Urea (mmol/l)	4.5 (3.4–5.5)	4.40 (3.2–5.33)	4.6 (3.6–5.9)	−1.437	0.15
3.6–9.5	119 (95.20)	63 (95.45)	56 (94.92)	0.000	1.000
>9.5	6 (4.80)	3 (4.55)	3 (5.08)		
Creatinine (μmol /l)	69.0 (57.0–87.0)	68.0 (55.75–88.25)	69.0 (60.0–88.0)	−0.646	0.519
59–104	114 (91.20)	61 (92.42)	53 (89.83)	0.261	0.609
>104	11 (8.80)	5 (7.58)	6 (10.17)		
LDH (U/L)	200 (165.5–269.5)	182.5 (153.75–232.75)	239.0 (190.0–303.0)	−3.793	0.000^*^
135–225	74 (59.20)	48 (72.73)	26 (44.07)	10.594	0.001^*^
>225	51 (40.80)	18 (27.27)	33 (55.93)		
Potassium (mmol/l)	4.06 (3.76–4.32)	4.07 (3.74–4.25)	4.04 (3.77–4.37)	−0.361	0.718
3.5–5.1	110 (88.00)	60 (90.91)	50 (84.75)	1.447	0.668
<3.5	13 (10.40)	5 (7.58)	8 (13.56)		
>5.1	2 (1.60)	1 (1.51)	1 (1.69)		
Sodium (mmol/l)	139.4 (136.9–140.7)	139.5 (137.8–140.75)	139.0 (134.8–140.7)	−1.521	0.121
136–145	102 (81.60)	59 (89.40)	43 (72.88)	7.667	0.021^*^
<136	22 (17.60)	6 (9.09)	16 (27.12)		
>145	1 (0.80)	1 (1.51)	0		
Chlorine (mmol/l)	101.1 (98.35–103.0)	101.85 (99.6–102.92)	100.0 (97.0–103.3)	−1.551	0.121
99–110	87 (69.60)	52 (78.79)	35 (59.32)	5.579	0.019^*^
<99	38 (30.40)	14 (21.21)	24 (40.68)		
Calcium (mmol/l)	2.16 (2.08–2.23)	2.19 (2.13–2.25)	2.12 (2.03–2.18)	−3.541	0.000^*^
2.2–2.5	44 (35.20)	32 (48.48)	12 (20.34)	10.819	0.001^*^
<2.20	81 (64.80)	34 (51.52)	47 (79.66)		
PT (sec)	13.60 (13.05–14.10)	13.5 (12.97–13.90)	13.8 (13.2–14.3)	−2.176	0.030^*^
11.5–14.5	112 (89.60)	63 (95.45)	49 (83.05)	5.143	0.023^*^
>14.5	13 (10.40)	3 (4.55)	10 (16.95)		
APTT (sec)	37.8 (35.7–40.9)	37.7 (35.6–40.9)	37.9 (36.3–41.6)	−0.480	0.631
29.0–42.0	102 (81.60)	57 (86.36)	45 (76.27)	2.113	0.146
>42.0	23 (18.40)	9 (13.64)	14 (23.73)		
TT (sec)	16.6 (15.85–17.2)	16.5 (15.8–17.12)	16.7 (16.0–17.6)	−1.205	0.228
14.0–19.0	119 (95.20)	64 (96.97)	55 (93.22)	0.958	0.420
>19.0	6 (4.80)	2 (3.03)	4 (6.78)		
Fibrinogen (g/L)	3.84 (2.98–5.1)	3.37 (2.73–4.49)	4.69 (3.22–5.78)	−3.665	0.000^*^
2–4	90 (72.00)	56 (84.85)	34 (57.63)	11.450	0.001^*^
>4	35 (28.00)	10 (15.15)	25 (42.37)		
D-dimer (ug/mL)	0.37 (0.22–0.83)	0.22 (0.22–0.78)	0.50 (0.26–1.01)	−2.760	0.006^*^
<0.5	75 (60.00)	45 (68.18)	30 (50.85)	3.900	0.048^*^
≥0.5	50 (40.00)	21 (31.82)	29 (49.15)		
**Chest CT findings**					
No pneumonia	11 (8.80)	9 (13.64)	2 (3.39)	16.845	0.00^*^
Unilateral pneumonia	53 (42.40)	36 (54.55)	17 (28.81)		
Bilateral pneumonia	61 (48.80)	21 (31.81)	40 (67.80)		
**Treatment**					
Antibiotics	80 (64.00)	33 (50.00)	47 (79.66)	11.895	0.001^*^
Antiviral	118 (94.40)	60 (90.90)	58 (98.31)	3.223	0.073
Oxygen therapy	72 (57.60)	36 (54.55)	36 (61.02)	3.206	0.201

Data are shown as median (interquartile range) or n (%). P-values were calculated by Mann-Whitney U test, χ^2^ test or Fisher's exact test, as appropriate. WBC, White blood cell; PLT, platelet count; CRP, C-reactive protein; BNP, B-type natriuretic peptide; CKMB, creatine kinase isoenzyme; ALT, alanine aminotransferase; AST, aspartate aminotransferase; LDH, lactic dehydrogenase; PT, prothrombin time; ATPP, activated partial thromboplastin time; TT, thrombin time; CT, computed tomographic;

* *P < 0.05 denoted siginificant difference between patients with short-term and long-term hospitalization*.

### Multivariable Analysis of Risk Factors Associated With Long-Term Hospitalization

**Figure 2:**
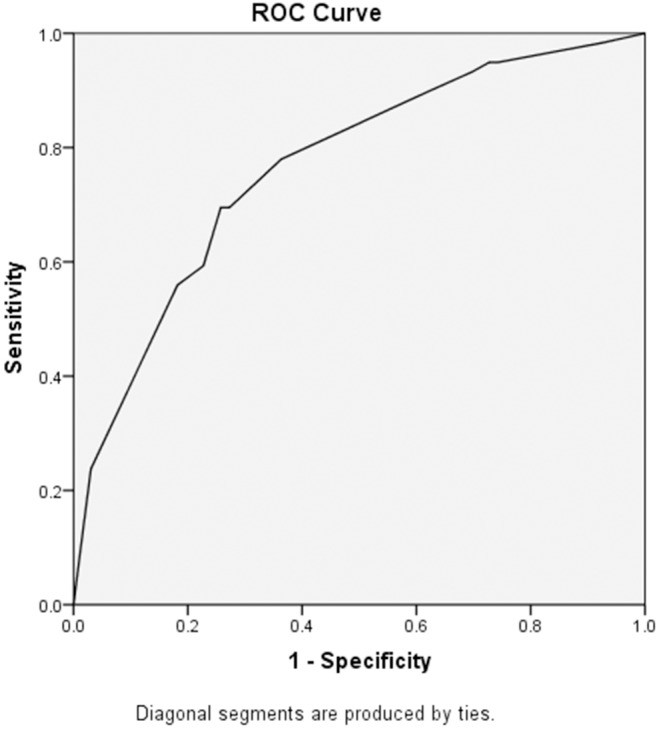
ROC curve of the multivariable model in the prediction of delayed discharge. The area under the ROC was 0.766 for retained variables in the multivariable model. With this multivariable model, more than three-quarters of the patients discharged ≥14 days could be classified correctly.

Multivariable analysis of risk factors associated with long-term hospitalization is shown in Table 3. Overall, 19 candidate variables with a *P* ≤ 0.10 in univariable analysis and two variables highly related to the outcome (age and comorbidity) were included in the multivariable model to identify risk factors associated with long-term hospitalization. The results indicated that hypocalcemia (*P* = 0.007, OR 3.313, 95% CI 1.392–7.886) and hypochloremia (*P* = 0.029, OR 2.663, 95% CI 1.104–6.621) on hospital admission were independent risk factors associated with long-term hospitalization in patients with COVID-19. Moreover, bilateral pneumonia showed in chest CT on hospital admission was also independently associated with long-term hospitalization, with an OR 5.907 (*P* = 0.009, 95% CI 1.073–32.521) compared with no pneumonia showed in chest CT and an OR 3.772 (*P* = 0.002, 95% CI 1.654–8.601) compared with unilateral pneumonia showed in chest CT. The Hosmer and Lemeshow test suggested that these variables included in this model could be well predictive for patients with long-term hospitalization (*P* = 0.985). Furthermore, a ROC curve where the area under the ROC was 0.766 for these retained variables is also presented (Figure 2). With this multivariable model, more than three-quarters of the patients discharged ≥14 days could be classified correctly.

**Table 3:** Multivariable analysis of risk factors associated with long-term hospitalization.

**Variables**	**Odds ratio**	**95% CI**	***P***
**Calcium (mmol/l)**			
2.2–2.5	1.0	Reference	
<2.20	3.313	1.392–7.886	0.007
**Chlorine (mmol/l)**			
99–110	1.0	Reference	
<99	2.663	1.104–6.621	0.029
**Chest CT findings**			
No pneumonia	1.0	Reference	
Unilateral pneumonia	1.566	0.280–8.772	0.610
Bilateral pneumonia	5.907	1.073–32.521	0.009
**Chest CT findings**			
Unilateral pneumonia	1.0	Reference	
Bilateral pneumonia	3.772	1.654–8.601	0.002
No pneumonia	0.639	0.114–3.577	0.610

## Discussion

In this retrospective study, almost half of the patients in this hospital ward were found to be discharged ≥14 days after hospital admission despite a long duration from symptom onset to present to the hospital. Theoretically, without effective anti-SARS-Cov-2 drugs, viral clearance may be the determining factor of the recovery time, especially for non-severe patients (18). In line with our result, a prior study indicated that the median hospitalization time was 16 days in 215 non-severe patients with COVID-19 from Shanghai, China (16). Moreover, the median onset-to-recovery time of the 125 patients was up to 30.0 days (IQR 21.5–43.0), which were longer than those in recently reported studies (10, 19). In view that delayed hospital admission after illness onset was found to be independently associated with a prolonged period of SARS-Cov-2 RNA shedding (20), the relatively long duration from illness onset to hospital admission might be a potential contributor to the longer onset-to-recovery time in our study.

Recently, several reports have shown that among patients with COVID-19, older patients and patients with any comorbidity had poorer clinical outcomes (3, 10, 17, 21). We performed a further analysis of risk factors associated with long-term hospitalization in patients with COVID-19. Surprisingly, age and comorbidity were not associated with long-term hospitalization in this study, although patients aged <60 years tended to have a shorter hospitalization time. It is difficult to explain this result, but it might be due to a small fraction of severe patients (2/125; 1.60%) in this study population. Moreover, abnormal coagulation parameters (e.g., prolonged prothrombin time, elevated levels of fibrinogen and D-dimer) and cardiac injury characterized by elevated levels of high sensitive troponin I, myoglobin, and lactic dehydrogenase were recently found to be related to poor outcomes in patients with COVID-19 (10, 22–25).

Even so, here, no significant statistical difference was found in the relationship between long-term hospitalization and these abnormal parameters in the multivariable logistic regression. This result may be explained by the fact that the median time from illness onset to hospital admission in this study was up to 15.0 days (IQR 7.0–30.0), which were much longer than those reported in other studies (2–6, 10, 12–16, 26). Take the coagulation parameters for instance, since coagulation is activated and accelerated as the first line of defense against acute infection (27), abnormal coagulation parameters tend to appear in the early stage of the disease course. Given this, the predictive performance of these abnormal parameters on the clinical outcome may be limited if the specimens were obtained in the middle or later stages of the disease course. Thus, a further study with more focus on this subject is therefore suggested.

It is interesting to note that a large proportion of patients had electrolyte imbalance including hypocalcemia (81/125; 64.80%), hypokalemia (13/125; 10.40%), hyponatremia (22/125; 17.60%), and hypochloremia (38/125; 30.40%), which was rarely mentioned in the current published research on COVID-19. A possible explanation for this result may attribute the long-term inadequate dietary intake of patients with COVID-19 owing to the long duration of illness before admission. Additionally, gastrointestinal symptoms such as vomiting, diarrhea, causing electrolyte imbalance, were also commonly reported (around 11 %) (26, 28). Unfortunately, the data of gastrointestinal symptoms in the 125 patients were missing. Similarly, electrolyte imbalance is also common in patients with severe acute respiratory syndrome (SARS) or Ebola virus disease (29, 30). For instance, 60% (53/89) of patients with SARS had hypocalcemia on hospital admission, close to the data in our study (29).

Moreover, our results showed that hypocalcemia (OR 3.313, 95% CI 1.392–7.886) and hypochloremia (OR 2.663, 95% CI 1.104–6.621) on hospital admission were independent risk factors associated with long-term hospitalization in patients with COVID-19. To the best of our knowledge, this is the first study to demonstrate the role of electrolyte balance in the hospitalization time of patients with COVID-19. There are several possible explanations for this result. First, it was suggested that calcium ions (Ca^2+^) play a pivotal role in membrane entry and fusion of coronavirus via a Ca^2+^ binding pocket with conserved glutamic acid (E) and aspartic acid (D) residues (31). Given this result, a lower calcium concentration might reflect a higher viral load when the human body is infected with a coronavirus, leading to a prolonged period of viral shedding. This provides an important focus of COVID-19 patients in future research. Second, prolonged hospitalization time is common in patients with a more serious condition and as such patients with higher severity of COVID-19 may have a longer hospitalization time (16). On the one hand, a systemic review demonstrated that a statistically lower calcium concentration was found in severe COVID-19 patients compared with non-severe patients (32), indicating that hypocalcemia might be related to higher severity of COVID-19. On the other hand, compared to COVID-19 patients without gastrointestinal symptoms, a higher severity tendency was observed in those with gastrointestinal symptoms, and patients with the more prone to an electrolyte imbalance caused by gastrointestinal symptoms trended toward the severe/critical type of the disease (28). Third, electrolyte concentrations such as calcium and chloride were reported to be related to the lung function and capacity of defense against invading pathogenic microorganisms in pulmonary infections (33, 34), suggesting that electrolyte imbalance might induce a delayed recovery from pulmonary infections. Finally, patients with electrolyte imbalance may need more hospitalization time to correct electrolyte abnormalities, compared with those without electrolyte imbalance. Thus, an essential strategy of the clinical management of COVID-19 is the availability of laboratory testing to closely monitor water-electrolyte status and acid-base balance. Besides, the used treatments should be reviewed not to have a significant effect on the electrolyte levels. Once such adverse reactions are suspected, more aggressive correction of electrolyte imbalance or discontinuation of suspect treatments should be considered as soon as possible.

Furthermore, bilateral pneumonia showed in chest CT on hospital admission was another independent risk factor associated with long-term hospitalization in COVID-19 patients compared with either unilateral pneumonia (OR = 3.772, 95% CI 1.654–8.601) or no pneumonia (OR = 5.907, 95% CI 1.073–32.521). These results match those observed in earlier studies which indicated that higher CT involvement scores including peripheral distribution and bilateral involvement were associated with the severity and mortality of COVID-19 patients (35–37), which commonly have an influence on hospitalization time. Additionally, one of the criteria of discharge for COVID-19 patients is that the acute exudative lesions showed in chest CT should improve significantly. Obviously, patients with bilateral pneumonia need more hospitalization time to meet this criterion. Therefore, chest CT findings can help not only in the evaluation of the severity but also in the prediction of the hospitalization time in COVID-19.

Despite the intriguing findings of our study, several important limitations should be taken into account. First, this study is single-centered research with a small sample size, and it may be underpowered to detect a significant difference between patients with short- and long-term hospitalization. Particularly, our results were obtained based on a standard statistical method instead of a state-of-the-art method (e.g., artificial intelligence). Artificial intelligence has been reported to can improve COVID-19 diagnosis and prediction (38). This is an important issue for future research on COVID-19. Second, owing to the retrospective study design, not all laboratory tests were performed in all patients, including CD_4_ T cell, CD_8_ T cell, and viral load. Hence, the role of these missing indicators might be underreported in the prediction of long-term hospitalization. In addition, the treatments for COVID-19 in this study were not the same for all patients, and whether the difference of treatments has an influence on the result is still unknown. Third, since the duration from illness onset to hospital admission was relatively long and varied widely among patients in this study, the value of hospitalization time in evaluating the onset-to-recovery time is limited. Especially, recall bias regarding symptoms at the illness onset was also great due to this long duration. Fourth, the period of viral shedding is crucial in the hospitalization time of patients, but it was not recorded in this study. Fifth, some COVID-19 patients may change to a positive result of SARS-Cov-2 again after discharge, and therefore the updated assessments and follow-up are important. However, only a follow-up of 2 weeks after discharge was conducted in the 125 patients, and it is unknown about the long-term outcomes of these patients. Thus, a large prospective cohort study with long-term follow-up is needed to verify our conclusions in the future. Last but not least, the time point of laboratory indices and radiographic findings was relatively late due to delayed hospital admission in these patients. Accordingly, this time point should be considered when our results are applied in predicting the hospitalization time of other patients. Further studies, which take these variables into account, will need to be undertaken.

## Conclusions

In summary, almost half of the patients were discharged ≥14 days after hospital admission despite a long duration from symptom onset to present to the hospital. Hypocalcemia, hypochloremia, and bilateral pneumonia on hospital admission were shown to be the independent risk factors associated with long-term hospitalization in patients with COVID-19. Our observations highlight the importance of electrolyte imbalance in predicting the hospitalization time of patients with COVID-19. Thus, special attention should be paid to the laboratory electrolyte results of the COVID-19 patients in clinical practice.

## Data Availability Statement

The raw data supporting the conclusions of this article will be made available by the authors, without undue reservation, to any qualified researcher.

## Ethics Statement

The studies involving human participants were reviewed and approved by the Ethics Committee of The Third Hospital of Xiamen Affiliated to Fujian University of Traditional Chinese Medicine. Written informed consent for participation was not required for this study in accordance with the national legislation and the institutional requirements.

## Author Contributions

PZ conceived and designed the research. YW, BH, JL, and YC collected the data and performed the research. Data were analyzed by PZ and YW. YW, BH, and PZ drafted the manuscript. PZ initiated and organized this study. All authors reviewed and edited the manuscript and approved the final version of the manuscript.

## Conflict of Interest

The authors declare that the research was conducted in the absence of any commercial or financial relationships that could be construed as a potential conflict of interest.
